# Mechanisms responsible for the vascular effect of aqueous *Trigonella foenum-graecum* leaf extract in diabetic rats

**DOI:** 10.4103/0253-7613.41039

**Published:** 2008

**Authors:** Mohammad Reza Vaez Mahdavi, Mehrdad Roghani, Tourandokht Baluchnejadmojarad

**Affiliations:** Department of Physiology, School of Medicine, Shahed University and Medicinal Plant Research Center, Tehran, Iran; 1 Department of Physiology, School of Medicine, Iran University of Medical Sciences, Tehran, Iran

**Keywords:** Aorta, diabetes mellitus, rat, streptozotocin, *Trigonella foenum-graecum*

## Abstract

**Background and Objective:**

Since a beneficial vascular effect of aqueous leaf extract of *Trigonella foenum-graecum* (TFG) has previously been reported, this study was conducted to evaluate the underlying mechanisms, including the role of nitric oxide (NO) and cyclooxygenase pathways, in diabetic rats.

**Materials and Methods:**

Male Wistar rats were divided into control, extract-treated control, diabetic, and extract-treated diabetic groups. Diabetes was induced by a single i.p. injection of streptozotocin (STZ; 60 mg/kg). Treatment groups received TFG extract (200 mg/kg; ip.) every other day for 1 month. Contractile reactivity of the thoracic aorta to KCl and noradrenaline (NA) and relaxation response to acetylcholine (ACh) were determined. For determination of the participation of NO and prostaglandins in the relaxation response to ACh, aortic rings were incubated for 30 min before the experiment with N-nitro-l-arginine methyl ester (L-NAME) and/or indomethacin (INDO).

**Results:**

The diabetic state significantly increased the maximum contractile response to KCl and NA (*P* < 0.01-0.005) and reduced the maximum relaxation due to ACh (*P* < 0.01) as compared to controls and treatment with TFG extract in the diabetic group significantly improved these changes relative to the untreated diabetic group (*P* < 0.05). With L-NAME pretreatment, no significant difference between diabetic and extract-treated diabetic groups was found out. On the other hand, there was a significant difference between these two groups following INDO pretreatment (*P* < 0.05).

**Conclusion:**

Intraperitoneal administration of aqueous leaf extract of TFG for one month could improve some functional indices of the vascular system in the diabetic state and endothelium-derived prostaglandins are essential in this respect.

Mortality from cardiovascular abnormalities, including hypertension, atherosclerosis, microangiopathy, and congestive heart failure, is almost three times higher in the diabetic population than in the general population.[1–2] Therefore, finding new treatment strategies for attenuation of diabetic vascular complications has always been an important aim in medicine. In this regard, *Trigonella foenum-graecum* (TFG; fenugreek) is considered a promising candidate. TFG is a plant with traditional medicinal use in diabetes. Beneficial effects have been demonstrated in diabetic animals and in both insulin-dependent and non-insulin-dependent diabetic subjects.[3] The hypoglycemic and antihyperglycemic effects of fenugreek seed and aqueous extracts of the leaf have previously been reported in experimentally induced diabetic rats.[4–5] In addition, endothelium-dependent attenuating effect of aqueous leaf extract of this plant on contractile reactivity of aortic rings from diabetic rats has previously been reported.[6] Therefore, the present study was carried out to evaluate the mechanisms responsible for its beneficial effect on the vascular reactivity of the thoracic aorta from STZ-diabetic rats.

## Materials and Methods

### Preparation of TFG extract

Fresh fenugreek was obtained from the local grocery (Tehran) in June and was systemically identified. The green leaves were separated, cleaned, and shade dried at room temperature. Then, 100 g of dried leaves was ground into a powder which was mixed with 1000 ml of boiling distilled water for a period of 10 min under continuous stirring. The obtained mixture was filtered twice through a mesh and the resultant liquid was dried in an evaporator until a concentrated residue (54% w/w; 21 g) was obtained. This stock extract was maintained at −20°C until it was used. Fenugreek extract of lower concentrations was prepared by dilution of the stock with cold and sterile 0.9% saline solution.

### Animal experiments

Male albino Wistar rats (Pasteur's institute, Tehran, Iran) 9-12 weeks old and weighing 240-280 g were used for the study. They were housed in an air-conditioned colony room at 23 ± 1°C and supplied with standard pellet diet and tap water *ad libitum*. Procedures involving animals and their care were conducted in conformity with the standard guidelines set out for the care and use of laboratory animals.

The animals (*n* = 30) were randomly divided into four experimental groups: vehicle-treated control (VC; *n* = 8), extract-treated control (EC; *n* = 7), vehicle-treated diabetic (VD; *n* = 7), and extract-treated diabetic (ED; *n* = 8) groups. Diabetes was induced by a single intraperitoneal injection of streptozotocin (STZ, 60 mg/kg) which was dissolved in cold 0.9% saline immediately before use. Vehicle-treated and extract-treated control animals received normal saline solution and aqueous extract of fenugreek (200 mg/kg, i.p.) respectively. The latter was administered one other day to extract-treated diabetic animals from day 3 after diabetes induction. Serum glucose level and body weight were measured 1 week before and 4 weeks after the study. Diabetes was verified by finding a nonfasting serum glucose level higher than 250 mg/dl using the glucose oxidation method (glucose oxidase kit, Zistchimie, Tehran, Iran). All treatments were continued for one month.

### Experimental procedure

The routine protocol was applied as described elsewhere.[7–8] Briefly, after the rats were anesthetized, the descending thoracic aorta was carefully excised and placed in a petri dish filled with cold Krebs solution containing (in mM): NaCl 118.5, KCl 4.74, CaCl_2_ 2.5, MgSO_4_ 1.18, KH_2_PO_4_ 1.18, NaHCO_3_ 24.9, and glucose 10.0.[7] The aorta was cleaned of excess connective tissue and fat and cut into rings of approximately 4 mm in length. Two rings from each aorta were left intact and in the other two rings, endothelium was mechanically removed by gently rotating them on a glass micropipette. The aortic rings were suspended between the bases of two triangular-shaped wires. One wire was attached to a fixed tissue support in a 50 ml isolated tissue bath containing Krebs solution (pH 7.4) maintained at 37°C and continuously aerated with a mixture of 5% CO_2_ and 95% O_2_. The other end of each wire was attached by a cotton thread to a F60 isometric force transducer (Narco, USA), which was connected to the A/D board of an IBM-compatible computer. Recording and analysis of data was performed using the software Physiograph I (Behineh Arman Co., Tehran, Iran). The rings were allowed to equilibrate for 60 min under a resting tension of 2 g before the experiments were begun. During the equilibration period, the rings were washed every 30 min. Successful removal of the endothelium was confirmed by loss of ACh (10^−5^ M)-induced relaxation in rings preconstricted by NA (10^−6^ M). Concentration-response curves were obtained first with KCl and then with NA in the aortic rings with and without endothelium. For this, KCl (10-50 mM) and NA (10^−9^-10^−4^ M) were added in a cumulative manner until a maximum response was achieved. For endothelium-intact and endothelium-denuded segments, concentration-dependent relaxation response to ACh (10^−9^-10^−4^ M) and sodium nitroprusside (SNP) (10^−9^-10^−4^ M), respectively, were obtained.

To determine the participation of NO in the relaxation response, the rings were incubated 30 min before the experiment with L-NAME (100 μM, a NOS inhibitor). To determine the participation of endothelial prostanoids in the relaxation response, the segments were incubated with INDO (10 μM, an inhibitor of COX-derived prostanoid synthesis) 30 min before the experiment.

After each experiment, the aortic rings were dried at 45°C for 5 min, weighed, and the cross-sectional area (CSA) was calculated using the following formula: cross-sectional area (mm^2^) = weight (mg) × [length (mm) × density (mg/mm^3^)]^−1^. The density of the preparations was assumed to be 1.05 mg/mm^3^.[9]

### Drugs and chemicals

Noradrenaline, ACh-HCl, and SNP were purchased from Sigma Chemicals (St. Louis, MO., USA). Streptozotocin was obtained from Pharmacia and Upjohn (USA). All other chemicals were purchased from Merck (Germany) and Temad (Tehran, Iran). STZ was freshly dissolved in 0.9% saline solution. Indomethacin solution was prepared in ethanol in such a way that the maximal ethanol concentration of the medium was less than 0.001% (v/v).

### Data and statistical analysis

All values are shown as mean ± SEM. Contractile responses to NA and KCl are expressed as grams of tension per cross-sectional area of tissue. Relaxation responses are shown as percentages. Statistical analysis was carried out using Student's paired t-test and one-way analysis of variance (ANOVA) followed by Tukey post-hoc test. A *P* value less than 0.05 is considered significant.

## Results

### Body weight, serum glucose, and cross-sectional area

Body weight and serum glucose level were measured before and at different weeks after the experiment [Table 1]. After 1 month, the body weight of the diabetic rats was found to be significantly lower as compared to the weight 1 week before the experiment (*P* < 0.05). Diabetic rats had significantly higher serum glucose level than the control rats (*P* < 0.001). Treatment of diabetic rats with aqueous extract of fenugreek (200 mg/kg) caused a significant lowering of serum glucose (*P* < 0.05). There was higher body weight in extract-treated diabetic rats as compared to the diabetic group, but this difference was not statistically significant. Furthermore, there was a significant reduction in cross-sectional area of the aortic rings in the diabetic group (*P* < 0.05) and the extract-treated diabetic group did not show any significant difference as compared to diabetics (data not shown).

**Table 1:** Body weight and serum glucose level of experimental groups

*Groups*	*Body weight (g)*		*Serum glucose (mg/dl)*	
		
	*Before*	*After*	*Before*	*After*
VC	268.3 ± 4.9*	279.6 ± 5.5	138.7 ± 4.8**	141.3 ± 4.6*
EC	271.6 ± 5.3*	265.3 ± 5.7	127.1 ± 5.4*	131.8 ± 5.6*
VD	274.3 ± 6.4*	219.8 ± 4.9**	142.3 ± 4.9*	389.1 ± 13.7***
ED	259.8 ± 5.4*	231.4 ± 6.2	141.2 ± 5.2	291.3 ± 11.7**#

* *P* < 0.05,

** *P* < 0.005,

*** *P* < 0.001 (in comparison with control);

# *P* < 0.05 (in comparison with diabetic). (VC, EC, VD, and ED represent vehicle-treated control, extract-treated control, vehicle-treated diabetic, and extract-treated diabetic rats, respectively)

### Contractile responses to KCl and NA

**Figure 1:**
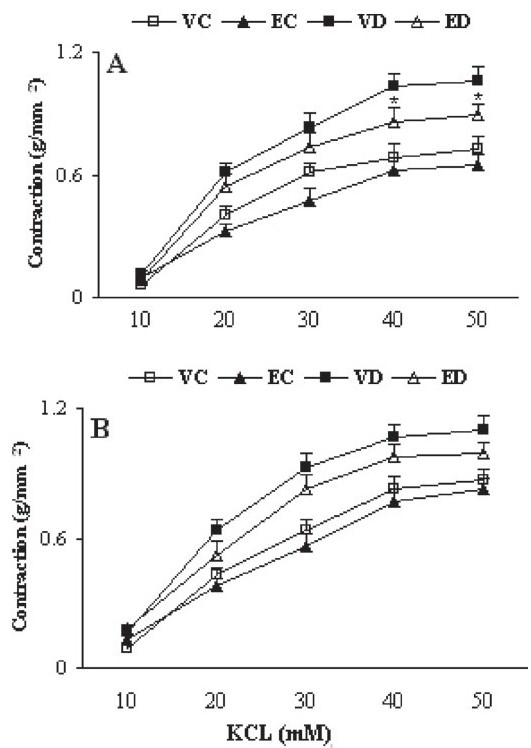
Cumulative concentration-response curves for KCl in aortic preparations after 1 month in the presence (A) or absence (B) of endothelium. Contractile responses are expressed as grams of tension per square millimeter of cross-sectional area (mm^2^). Data are shown as means ± SEM. *P < 0.05 (as compared to VD) (VC, EC, VD, and ED represent vehicle-treated control, extract-treated control, vehicle-treated diabetic, and extract-treated diabetic rats, respectively)

**Figure 2:**
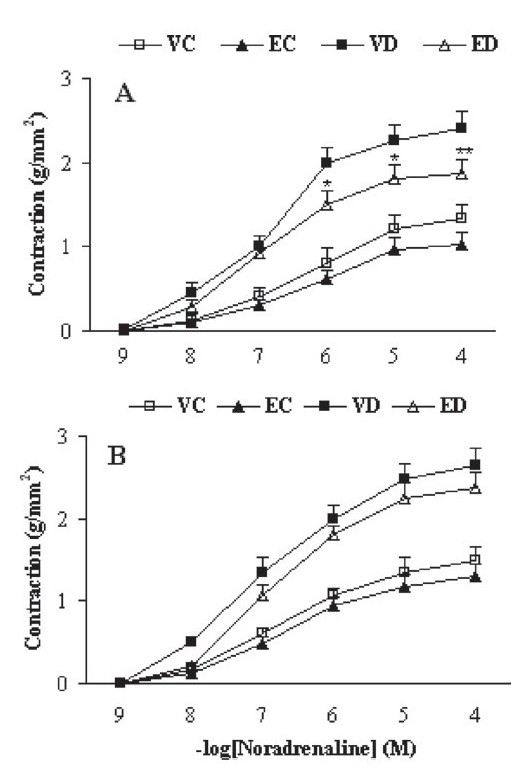
Cumulative concentration-response curves for NA in aortic preparations after 1 month in the presence (A) or absence (B) of endothelium. Contractile responses are expressed as grams of tension per square millimeter of cross-sectional area (mm^2^). Data are shown as means ± SEM. *P < 0.05, **P < 0.01 (as compared to VD) (VC, EC, VD, and ED represent vehicle-treated control, extract-treated control, vehicle-treated diabetic, and extract-treated diabetic rats, respectively)

Cumulative addition of KCl (10-50 mM) and NA (10^−9^ 10^−4^ M) to the organ bath resulted in concentration-dependent contractions in the aortas of all groups [Figures 1 and 2]. The contractile responses to KCl at concentrations higher than 20 mM in diabetic rats were found to be significantly higher than in control rats, both in the presence [Figure 1A] and absence of endothelium [Figure 1B]. Treatment of diabetic rats with TFG caused a significant reduction in the contractile response to KCl at concentrations higher than 40 mM only in endothelium-intact rings. The contractile responses to NA at concentrations higher than 10^−7^ M in vehicle-treated diabetic rats were found to be significantly higher than in vehicle-treated control rats, both in the presence [Figure 2A] and absence of endothelium [Figure 2B]; treatment of diabetic rats with TFG caused a significant reduction in the contractile response to NA only in endothelium-intact rings. Furthermore, treatment of control rats with TFG leaf extract did not produce any significant changes in response to KCl and NA in both endothelium-intact and endothelium-denuded aortic rings.

**Figure 3:**
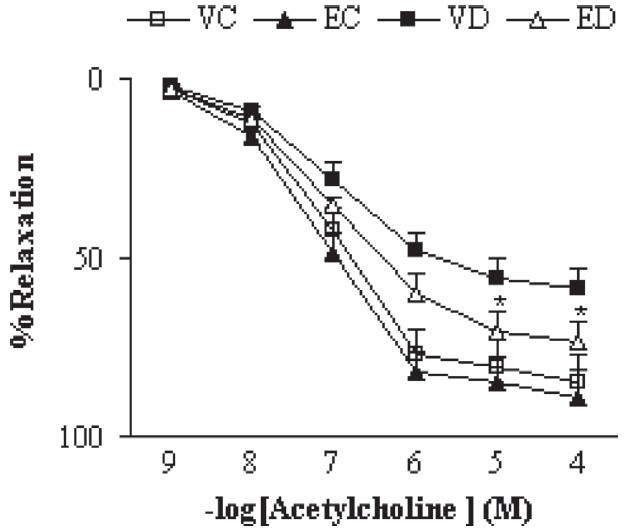
Cumulative concentration-response curves for ACh in NA-precontracted aortic preparations after 1 month. Relaxation response is expressed as percentage. Data are shown as means ± SEM. *P < 0.05 (as compared to VD) (VC, EC, VD, and ED represent vehicle-treated control, extract-treated control, vehicletreated diabetic, and extract-treated diabetic rats, respectively)

**Figure 4:**
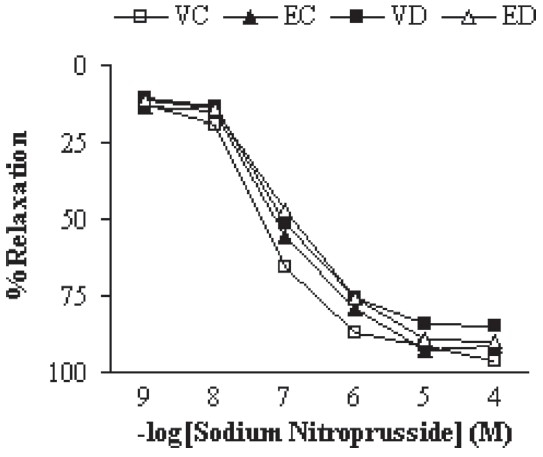
Cumulative concentration-response curves for SNP in NA-precontracted endothelium denuded aortic preparations after 1 month. Relaxation response is expressed as percentage. Data are shown as means ± SEM. (VC, EC, VD, and ED represent vehicle-treated control, extract-treated control, vehicle-treated diabetic, and extract-treated diabetic rats, respectively)

Addition of ACh resulted in concentration-dependent relaxations in all aortic rings precontracted with NA [Figure 3]. As was expected, endothelium-dependent relaxation responses induced by ACh were significantly lower in vehicle-treated diabetic rats as compared to vehicle-treated controls (*P* < 0.01). The existing difference between extract-treated and vehicle-treated diabetic rats was only significant (*P* < 0.05) at concentrations higher than 10^−5^ M. The relaxation response of extract-treated control rats was not significantly different from that of the control group. The endothelium-independent relaxation responses for SNP were also found not to be significantly different among the groups [Figure 4], indicating the importance of the presence of endothelium for deriving the beneficial effect of this extract.

**Figure 5:**
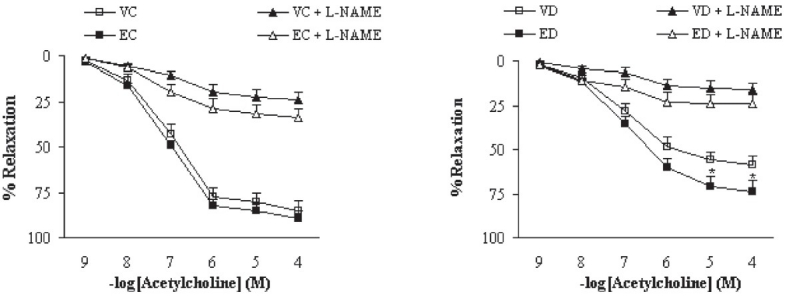
Cumulative concentration-response curves for ACh in NA-precontracted aortic preparations after 1 month in the absence and presence of L-NAME (at a concentration of 100 μM 30 min before the experiment). Relaxation response is expressed as percentage. Data are shown as means ± SEM. *P < 0.05 (as compared to VD) (VC, EC, VD, ED, and L-NAME represent vehicle-treated control, extract-treated control, vehicle-treated diabetic, extract-treated diabetic, and N-nitro-l-arginine methyl ester, respectively)

**Figure 6:**
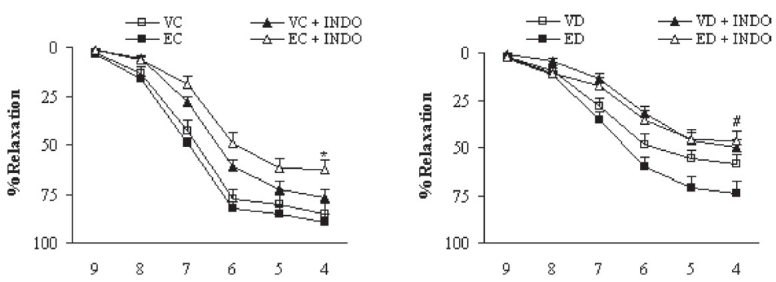
Cumulative concentration-response curves for ACh in NA-precontracted aortic preparations after 1 month in the absence and presence of indomethacin (at a concentration of 10 μM 30 min before the experiment). Relaxation response is expressed as percentage. Data are shown as means ± SEM. *P < 0.05 (EC + INDO *vs* EC); ^#^*P* < 0.05 (ED + INDO *vs* ED) (VC, EC, VD, ED, and INDO represent vehicle-treated control, extract-treated control, vehicle-treated diabetic, extract-treated diabetic, and indomethacin, respectively)

Preincubation of aortic rings with L-NAME almost completely abolished the vasodilator response to ACh in the segments from extract-treated control and diabetic rats [Figure 5]. The existing differences between control and extract-treated control and between diabetic and extract-treated diabetic groups were not statistically significant, indicating that the effect of TFG extract on the aorta is not due to induction of basal NO release. On the other hand, preincubation of aortic segments from extract-treated control and diabetic rats with INDO diminished the endothelial vasodilator response to ACh [Figure 6]. In this case, the differences between control and extract-treated control and between diabetic and extract-treated diabetic groups were statistically significant (*P* < 0.05), indicating that the effect of TFG extract on the aorta is partly due to enhanced basal activity of cyclooxygenase and its related prostanoid synthesis pathway with its predominance of vasorelaxant factors.

## Discussion

The results of the present study demonstrate that aortas from 1-month STZ-diabetic rats are more responsive to the contractile effect of the α-adrenoceptor agonist NA and to the nonspecific agent KCl, both in the presence and absence of endothelium, than those from corresponding controls. Similar results showing the increased vascular responsiveness to contractile agents in STZ-diabetic rats have been reported in many previous studies.[9–10] This increased vascular smooth muscle responsiveness in diabetic rats could be attributed to deficient endothelial activity,[9–10] enhanced phosphoinositide (PI) metabolism,[11] enhanced sensitivity of calcium channels,[7] and increased sensitivity to adrenergic agonists.[12] Furthermore, oxidative stress is increased due to excessive production of oxygen free radicals and decreased antioxidant defense systems.[13–15]

In this study, TFG attenuated the increased responsiveness of aortic rings in the diabetic state. Our results demonstrate that fenugreek extract at a dose of 200 mg/kg could partially counteract the increased contractile response of endothelium-intact aortic rings of diabetic rats following NA and/or KCl and could enhance the relaxation response in diabetic rats to Ach, which is consistent with previous reports.[6] The beneficial effect of chronic fenugreek extract treatment on NA- and KCl-induced contractions was specific for aortas of diabetic rats, because the extract treatment did not produce any significant change in control preparations.

The results of this study also show that the beneficial effect of TFG extract on the aorta of diabetic rats is partly mediated through the pathway of prostaglandin synthesis and there is no significant change in the basal capacity of endothelium to release NO. As indicated by the results of other studies, in the presence of INDO, the increased vasoconstrictor prostanoid release and decreased vasorelaxant prostanoid release is potentially an important mechanism in the endothelial dysfunction induced by diabetes and this abnormality is associated with different cardiovascular risk factors.[16–17]

To conclude, the findings of this study indicate that chronic treatment of diabetic rats with aqueous leaf extract of TFG could partially prevent the development of changes in vascular reactivity, and endothelial prostaglandins are essential in this respect.
